# The effect of SGLT-2 inhibitors on cardiorespiratory fitness capacity: A systematic review and meta-analysis

**DOI:** 10.3389/fphys.2022.1081920

**Published:** 2023-01-10

**Authors:** Yong Peng, Di Qin, Yudi Wang, Lian Xue, YaXuan Qin, Xin Xu

**Affiliations:** ^1^ School of Kinesiology, Shanghai University of Sport, Shanghai, China; ^2^ Jiangsu Collaborative Innovation Center for Sports and Health Project, Nanjing Sport Institute, Nanjing, Jiangsu, China; ^3^ Key Laboratory of Human Sports Science for Jiangsu Province, Nanjing Sport Institute, Nanjing, Jiangsu, China; ^4^ School of Sport Health, Nanjing Sport Institute, Nanjing, Jiangsu, China; ^5^ School of Physical Education and Nursing, Chengdu College of Arts and Sciences, Chengdu, China

**Keywords:** SGLT-2 inhibitors, exercise capacity, T2DM, heart failure, VO_2_peak

## Abstract

**Objective:** The study aimed to evaluate the effect of sodium–glucose transporter 2 (SGLT-2) inhibitors on various parameters of exercise capacity and provide an evidence-based basis for type 2 diabetes mellitus (T2DM) combined with heart failure (HF) patients or HF patients without T2DM who use SGLT-2 inhibitors to improve cardiorespiratory fitness (CRF).

**Methods:** According to the participant, intervention, comparison, and outcome (PICO) elements, the effects of SGLT-2 inhibitor administration on VO_2_ or VO_2_peak were researched in this study. Weighted mean difference (WMD) and 95% confidence intervals (CIs) were calculated (random-effects model). Heterogeneity was assessed by the I^2^ test.

**Results:** Six studies were included according to the eligibility criteria: four were RCTs, and two were non-RCTs. Compared with the control group, the merge results of RCTs showed that SGLT-2 inhibitors could significantly increase the VO_2_peak (WMD, 2.02 ml kg^−1^ min^−1^, 95% CI: 0.68–3.37, and *p* = 0.03; I^2^ = 0% and *p* = 0.40) and VAT (WMD, 1.57 ml kg^−1^ min^−1^, 95% CI: 0.06–3.07, and *p* = 0.04; I^2^ = 0% and *p* = 0.52) of the obese population, patients with T2DM, and chronic HF patients with or without T2DM. Subgroup analysis showed that SGLT-2 inhibitors improved the VO_2_peak in non-HF patients (WMD, 3.57 ml kg^−1^ min^−1^, 95% CI: 0.87–6.26, and *p* = 0.009; I^2^ = 4% and *p* = 0.31) more than in HF patients (WMD, 1.46 ml kg^−1^ min^−1^, 95% CI: −0.13–3.04, and *p* = 0.07; I^2^ = 0% and *p* = 0.81). Moreover, the merge of single-arm studies also indicated that empagliflozin could improve VO_2_peak (MD, 1.11 ml kg^−1^ min^−1^, 95% CI: 0.93–1.30, and *p* = 0.827, Δ *p* = 0.000 and I^2^ = 0%) of T2DM patients with chronic HF.

**Conclusion:** Despite the limited number of studies and samples involved, the meta-analysis preliminarily demonstrated that SGLT-2 inhibitors could improve some parameters of exercise capacity (VO_2_peak, VAT) in chronic HF patients with or without T2DM and obese individuals, which had a positive effect on promoting cardiopulmonary fitness to help these populations improve their prognosis.

**Systematic Review Registration:** [https://www.crd.york.ac.uk/prospero/#recordDetails], identifier [CRD42020202788].

## 1 Introduction

Type 2 diabetes mellitus (T2DM) is one of the most common chronic metabolic diseases and the major risk factor for cardiovascular diseases (CVDs). CVD is secondary to T2DM, which is the main cause of death, and HF is one of the most serious complications with adverse prognosis (Rao Kondapally Seshasai et al., 2011). Cardiorespiratory fitness (CRF) is one of the strongest predictors of mortality (Wei et al., 2000; Kokkinos et al., 2022). CVD patients or healthy people with a low level of CRF have higher risk ratios of all-cause mortality. A previous study indicated that individuals with low CRF (maximal aerobic capacity <7.9 METs) had 1.56-fold and 1.47-fold increased risk of CVD events, respectively, compared with those who had intermediate (7.9–10.8 METs) and high (≥10.8 METs) CRF (Kodama et al., 2009). In addition, some studies demonstrated that CRF and grip strength have a strong inverse association with HF incidence (Sillars et al., 2019). VO_2_ and VO_2_peak parameters had important prognostic values in HF (Popovic et al., 2018; Paolillo et al., 2019). Therefore, the core purpose of T2DM treatment is to generate cardiovascular benefits based on achieving a glycemic control target.

Exercise therapy combined with nutrition management is an effective treatment strategy for T2DM. In addition, most patients with diabetes also need hypoglycemic drugs to help them improve glycemic control and cardiovascular function (Samocha-Bonet et al., 2018). Metformin was recommended by clinicians as the first choice of treatment for patients with T2MD (Flory and Lipska, 2019). Previous literature reports demonstrated that metformin has beneficial effects on glucose homeostasis control and CVD (Andersson et al., 2010; Elder et al., 2016). Whether metformin improves cardiorespiratory endurance or exercise capacity remains unknown. However, there were complex potential links between exercise capacity and metformin. A meta-analysis showed that metformin did not affect exercise capacity parameters (VO_2_, VO_2_peak, and VAT) in healthy individuals, individuals with diabetes, and individuals with insulin resistance (Das et al., 2018a). Previous research indicated that exercise combined with metformin was not superior to exercise alone in improving VO_2_ in insulin-resistant individuals. Metformin combined with exercise therapy did not show further improvement in cardiopulmonary endurance in patients with insulin resistance or T2DM compared to exercise alone (Cadeddu et al., 2014). Therefore, individuals with T2DM combined with HF also needed second-line anti-diabetes agents, except for metformin.

Sodium–glucose transporter 2 (SGLT-2) inhibitors are novel hypoglycemic agents that selectively act on the renal sodium–glucose cotransporter 2, inhibit the glomerular reabsorption of glucose, and increase urine glucose to control the level of blood glucose (Kario et al., 2018). In addition, SGLT-2 inhibitors can help lose weight, decrease blood pressure, and reduce the risk of hypoglycemia in patients with T2DM (Kario et al., 2018). Notably, a growing number of clinical studies demonstrated that SGLT-2 inhibitors could decrease the risk of HF, worsening hospitalization, myocardial infarction, and stroke and significantly reducing the risk of CVD death and all-cause death (Zinman et al., 2015; McMurray et al., 2019; Wiviott et al., 2019). The guidelines and consensus on T2DM management issued were developed by the American Diabetes Association (ADA), American College of Cardiology (ACC), the ADA, and the European Association for the study of Diabetes (EASD), respectively, which all clearly suggested patients with T2DM combined with atherosclerosis cardiovascular disease (ASCVD) or high cardiovascular risk to take SGLT-2 inhibitors on the basis of using metformin (Das et al., 2018b; American Diabetes Association, 2020; Buse et al., 2020). With the widespread application of SGLT-2 inhibitors in clinics, their protective effect on the cardiovascular system of T2DM patients has been paid more attention. However, the effect of SGLT-2 inhibitors on exercise capacity or CRF in patients with T2DM combined with CVD was unknown, and no meta-analysis has indicated the effect of SGLT-2 inhibitors on exercise capacity so far. Therefore, the focus of this meta-analysis was to evaluate the effect of SGLT-2 inhibitors on the various parameters of exercise capacity in the obese population, patients with T2DM, and chronic HF patients with or without T2DM to indicate the potential relationship between SGLT-2 inhibitors and CRF and provide adequate evidence to support the cardiovascular protective benefits of SGLT-2 inhibitors in T2DM patients.

## 2 Methods

### 2.1 Search strategy

The Preferred Reporting Items for Systematic Reviews and Meta-Analyses (PRISMA) guideline specification was applied to this meta-analysis (Shamseer et al., 2015). English was used as the retrieval language, and the relevant articles on randomized controlled trials (RCTs) were searched from PubMed/MEDLINE, Web of Science, Ebsco CINAHL, Embase, and Cochrane Library databases. The search time range was limited from the latest available date to 1 August 2022. The search terms used were “SGLT2” or “SGLT2 inhibitor” or “Sodium glucose cotransporter 2 inhibitor” or “dapagliflozin” or “canagliflozin” or “empagliflozin” or “ipragliflozin” or “luseogliflozin” or “tofogliflozin” in combination with “cardiorespiratory fitness” or “cardiopulmonary endurance” or “endurance” or “exercise” or “exercise capacity” or “exercise tolerance” or “aerobic” or “aerobic exercise” or “oxygen” or “oxygen consumption” or “VO_2_” or “peak VO_2_” or “VO_2_max” or “VO_2_peak”. Other potentially relevant articles were selected from the reference lists of included articles.

### 2.2 Inclusion criteria

According to the participant, intervention, comparison, and outcome (PICO) elements, the eligibility criteria for inclusion were determined. The inclusion criteria were as follows: 1) types of studies: RCTs or non-RCTs that evaluated the effect of SGLT-2 inhibitor intervention on cardiopulmonary endurance were included, with no language restrictions of the included literature; 2) types of participants: patients were diagnosed with T2DM, chronic HF, or obesity (age ≥ 18, both gender) in accordance with the World Health Organization (WHO) or ADA diagnostic criteria with no limitation to gender, population, course of diseases, and complications; 3) types of interventions: the intervention measures of the experimental group were SGLT-2 inhibitors as single-drug treatment or SGLT-2 inhibitors combined with other antidiabetic agents on the basis of exercise combined with diet intervention; the interventions of the control group were placebo or other hypoglycemic drugs; and 4) types of outcome measures: VO_2_peak was taken as the primary outcome, while the respiratory exchange ratio (RER), VE/VCO_2_ slope (minute ventilation/carbon dioxide production), and VAT (ventilator anaerobic threshold) exercise capacity parameters were taken as the secondary outcomes to evaluate the effect of SGLT-2 inhibitors on exercise capacity. The exclusion criteria were age < 18 and any other diseases that could interfere with exercise capacity, except for T2DM, insulin resistance, metabolic syndrome, and HF, among others.

### 2.3 Data extraction and quality assessment

According to the predefined inclusion criteria, two reviewers read the title, abstract, and full text of the articles independently and then screened and determined whether they could be included. Two reviewers independently extracted data from the included articles that contained information about study characteristics, subject characteristics, study design, and outcomes measured with predefined criteria. If there were disagreements in data processing, they were resolved by discussion, or a third reviewer decided whether they should be included. The Cochrane Collaboration tool was used to assess the risk of bias in the included RCT studies (Higgins et al., 2011). The evaluation criteria that were unclear or low or had a high risk of bias were used to assess the methodological quality of each trial. We used the methodological index for non-randomized studies (MINORS) tool to assess the bias risk of non-RCT trials (Slim et al., 2003). Two reviewers independently assessed it, and if there were any disagreements, a third reviewer determined the final outcome of bias risk.

### 2.4 Statistical data

The data analysis of meta-analysis was conducted using Review Manager (RevMan 5.3) and Stata 12.0 software packages, and a random effects model was used to calculate higher heterogeneity. As primary or secondary outcomes were continuous variables, the changes between baseline and post-SGLT-2 inhibitors were used for this meta-analysis. We used the weighted mean difference (WMD) and 95% confidence intervals (CIs) to represent the main treatment effects, with *p* < 0.05 statistically significant. Heterogeneity among the studies was assessed by Cochran’s Q-test (*p* < 0.1) and quantified with the I^2^ test (I^2^ < 25%, 30% < I^2^ < 50%, and I^2^ > 50% were considered as minimal heterogeneity, moderate heterogeneity, and substantial heterogeneity, respectively). Sensitivity analysis was performed by deleting each research individually to evaluate the consistency and quality of the results.

## 3 Result

### 3.1 Included study characteristics

**FIGURE 1:**
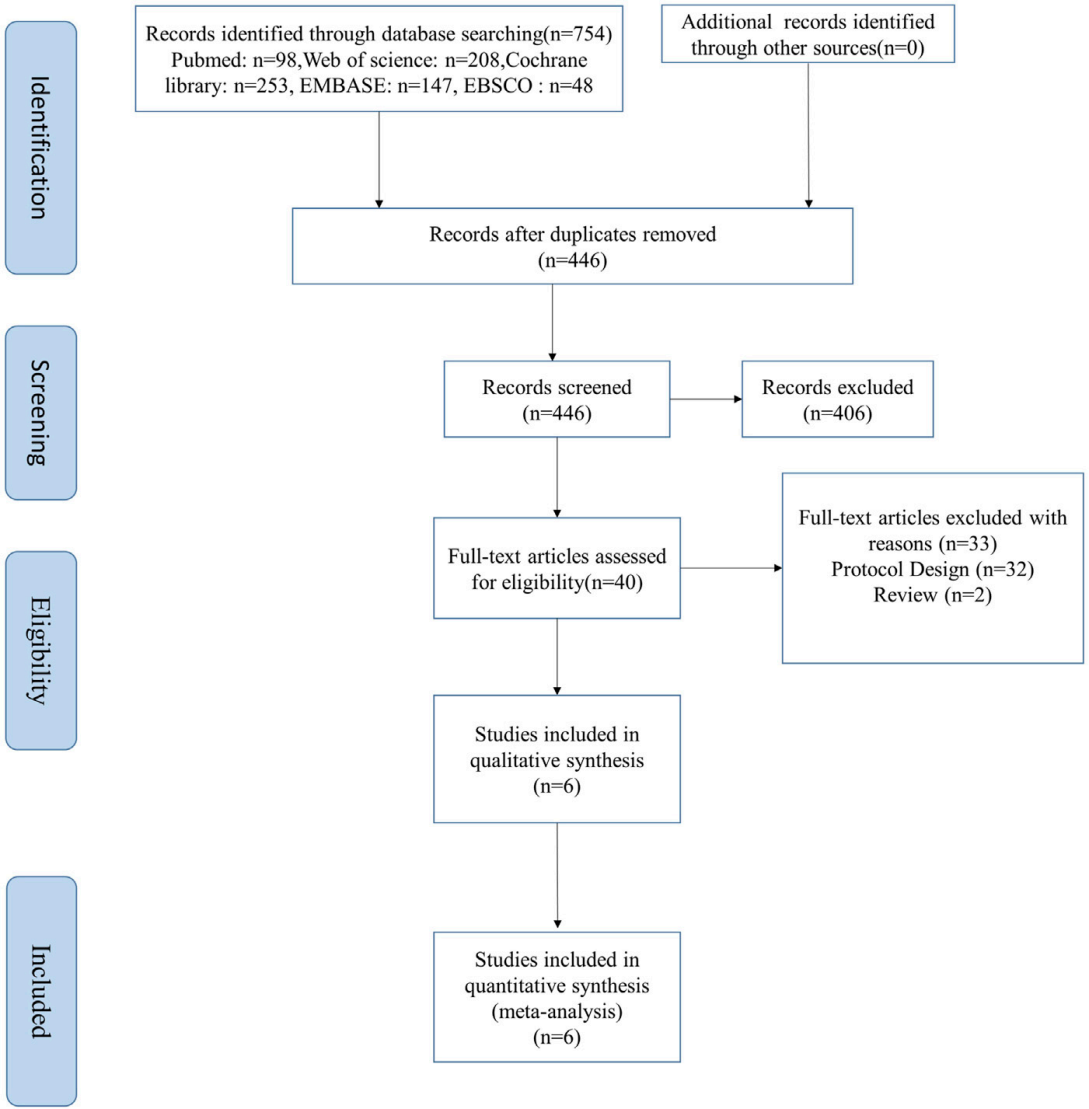
Flow diagram of study selection.

A total of 446 studies were searched from five English databases, and we finally selected 406 studies as the potentially eligible studies based on the title and abstract. After reviewing the full text, six studies were included in this meta-analysis, four of which were RCTs (Kumar et al., 2018; Newman et al., 2019; Carbone et al., 2020a; Santos-Gallego et al., 2020), and two, non-RCTs (Carbone et al., 2018; Núñez et al., 2018). The flow diagram of the studies is shown in Figure1. The total number of patients in the included four RCT studies was 175, with 84 patients in the SGLT-2 inhibitor group and 91 in the placebo or control drug group, respectively. In addition, another two non-RCT studies recruited 34 patients, and all the studies were small sample trials. The age of the patients was 54–79 years, except for those who were overweight and obese at 18–50 years in Newman’s study. They all had chronic HF and high risk for CVD with or without T2DM (New York Heart Association classes II–III).

Four studies were from the United States (Carbone et al., 2018; Newman et al., 2019; Carbone et al., 2020a; Santos-Gallego et al., 2020), and the rest, from Canada and Spain (Kumar et al., 2018; Núñez et al., 2018). Most of the studies used empagliflozin (Carbone et al., 2018; Kumar et al., 2018; Núñez et al., 2018; Santos-Gallego et al., 2020), whereas Newman et al. (2019) used dapagliflozin, and another study by Carbone used canagliflozin as the treatment drugs (Carbone et al., 2020a). The empagliflozin dose is 10 mg/d, and the dapagliflozin dose is less than or equal to 10 mg/d. However, the canagliflozin dose is 100 mg/d. The single-arm studies (Carbone et al., 2018; Núñez et al., 2018) used empagliflozin for 1 month, whereas the duration of drugs in the other four studies ranged from 3 to 6 months (Kumar et al., 2018; Newman et al., 2019; Carbone et al., 2020a; Santos-Gallego et al., 2020). In addition, endurance exercise and SGLT-2 inhibitors were used as the combination treatment protocol to investigate the effect of SGLT-2 inhibitors on physiological adaptation to endurance exercise training (Newman et al., 2019). All studies used different measurement devices and procedures to evaluate outcomes of exercise capacity parameters before and after treatment. The cardiopulmonary exercise examination (CPX) test evaluated the exercise capacity parameters using a treadmill and gas metabolizer (Carbone et al., 2018; Carbone et al., 2020a), whereas other researchers used a cycle ergometer. The characteristics of subjects, intervention protocols, and outcomes in all included studies are summarized in Tables 1).

**TABLE 1:** Characteristics of the included studies.

Author, Year (Country/region)	Type of study	Participant Characteristic			Experimental Group	Control Group	Measurement of exercise capacity	Outcomes
		Age	Gender Male%	condition				
Newman et al. (2019) (USA)	Randomized controlled trial	EG:28 ± 12, CG:24 ± 10 Range:18 to 50 years	EG:26% (*n* = 15), CG:46.6% (*n* = 15)	sedentary overweight and obese men and women	Dapagliflozin ≤10 mg/day, 12 weeks (5 mg/day for the first 14 days). Other treatment: Endurance exercise training: treadmill walking, running, cycle ergometer exercise and elliptical ergometer exercise (70%–80% of heart rate reserve)	Placebo and Endurance exercise training	Cycle ergometer (continuous progressive exercise: External work were 25, 50, 100 W, every grade load continuous 10 min)	Primary outcomes: V_2_ peak, RER, fasting blood glucose, insulin sensitivity. Secondary outcomes: body composition, skeletal muscle citrate synthase activity, HR
Carbone et al. (2020) (USA)	Randomized controlled trial	EG:58± 6.1 CG:54.3 ± 8.8	EG:76.5% (*n* = 17), CG:78.9% (*n* = 19)	T2MD and stable chronic HF patients (New York Heart Association class Ⅱ–Ⅲ, LVEF ≤40%)	Canagliflozin 100 mg daily for 12 weeks	Sitagliptin 100 mg daily for 12 weeks	CPX Test (Treadmill continuous progressive exercise: the speed and grade increased approximately 0.6 METs/min	Primary outcomes: VO_2_ peak, VE/VCO_2_ slope. Secondary outcomes: Quality of life, VAT, body composition, blood pressure, HbA1c
Kumar et al. (2018) (Canada)	Randomized controlled trial	EG: 67.3 ± 8 CG: 66.4 ± 10	EG:90% (*n* = 10), CG:100% (*n* = 10)	Subjects with T2MD, and have high risk for cardiovascular disease.	Empagliflozin 10 mg daily for 6 month	Usual care receive standard medical therapy for diabetes and cardiovascular risk	Cycle ergometer (continuous progressive exercise: customized linear–ramp protocol designed to elicit fatigue within 8 – 12 min of exercise)	Primary outcomes: VO_2_ peak, VE/VCO_2_ slope. Secondary outcomes: HRR, RER, AT
Santos-Gallego et al. (2020) dDX(USA)	Randomized controlled trial	EG:62.4 ± 12.1 CG:59.9±13.1	EG:63% (*n* = 27), CG:64% (*n* = 27)	No–diabetic patients with heart failure (NYHA Ⅱ–Ⅲ, LVEF <50%)	Empagliflozin for 6 month	Statin, B–blockers, loop diuretics, Thiazide diuretics, mineralocorticoid antagonists, ca–blockers, antiplatelet, anticoagulants	CPET on a cycle ergometer, exercise began with unloaded exercise and increased by 25 watts every 3 minutes, respiratory exchange ratio 1.1 or Borg scale scores was at least 15, termination test	Primary outcomes: LVEDV, LVESV, CMR. Secondary endpoints: peak VO_2_, VE/VCO_2_, OUES,6MWT, KCCQ-12
Núñez et al. (2018) (Spain)	Non-Randomized controlled trial	Median age, 72(60–79)	73.7% (*n* = 19)	T2DM patients with chronic HF (LVEF <40%)	Empagliflozin 10 mg daily without changes in concomitant medications for 30 days		CPET on a bicycle ergometer (10-w is the beginning workload, every 1 min increase 10-w workload)	Primary outcomes: VO_2_ peak, Secondary outcomes: VE/VCO_2_ slope, 6MWT, MLHF
Carbone et al. (2018) (USA)	Non-Randomized controlled trial	Median age, 60(56–62)	47% (*n* = 15)	Patients with T2DM and stable symptomatic HFrEF (LVEF <50%,	empagliflozin 10 mg daily for four weeks	loop diuretics+	CPX Test (Treadmill continuous progressive exercise: speed and grade were increased by 0.6 MET every 60 s	Primary outcomes: VO_2_ peak, Secondary outcomes: fasting glycemia, blood pressure, Doppler echocardiography

Abbreviations: EG, experimental group; CG, control group; T2DM, type II diabetes mellitus; HF, heart failure; HFrEF, heart failure with reduced ejection fraction; LVEF, left ventricular ejection fraction; MET, metabolic equivalent of energy; CPX, cardiopulmonary exercise testing; CPET, cardiopulmonary exercise test; VO_2_, oxygen consumption; RER, respiratory exchange ratio; HR, heart rate; HRR, heart rate recovery; VE/VCO_2_, minute ventilation/carbon dioxide production; AT, anaerobic threshold; VAT, ventilatory anaerobic threshold; 6MWT, 6-minute walk test; MLHF, Minnesota Living with Heart Failure; LVEDV, left ventricular end-diastolic volume; LVESV, left ventricular end-systolic volume; CMR, cardiac magnetic resonance; KCCQ-12, Kansa city cardiac questionnaire-12; OUES, oxygen uptake efficiency slope.

### 3.2 Literature quality assessment

We used the Cochrane Collaboration tool to assess the methodological quality of the included RCT studies. There was a low-to-medium risk of bias in Newman and Carbone studies because of a lack of information about other biases. Kumar’s study did not specify the randomization method, which had a high risk of bias Table 2. In addition, the methodological qualities of all single-arm studies were assessed by the MINORS tool, and the total MINORS scores of Núñez and Carbone studies were 9 and 8, respectively Table 3. Their studies showed a high risk of bias of inadequate follow-up, a lack of blind evaluation of endpoint indicators, and a lack of prospective of the study size.

**TABLE 2:** Risk of bias assessment of included RCT studies by the Cochrane Collaboration tool.

Author, year	Random sequence generation	Allocation concealment	Blinding of participants and personnel	Blinding of outcome assessment	Incomplete outcome data	Selective reporting	Other biases
Newmanet al.(2019)	Low	Low	Low	Low	Low	Low	Unclear
Carboneet al.(2020)	Low	Low	Low	Low	Low	Low	High
Kumaret al.(2018)	High	High	High	High	Low	Low	Unclear
Santos-Gallego et al. (2020)	Low	Low	Low	Low	Low	Low	Unclear

**TABLE 3:** Risk of bias assessment of included non-RCT trials by the MINORS tool (0 score, not reported; 1 score, reported but not fully informed; 2 scores, reported and fully informed).

Author, year	Clearly stated aim	Inclusion of consecutive patients	Prospective collection of data	Endpoints appropriate to the aim of the study	Unbiased assessment of the study endpoint	Follow-up period appropriate to the aim of the study	Loss to follow-up less than 5%	Prospective of the study size	MINORS score
Núñez et al. (2018)	2	2	2	2	0	1	0	0	9
Carbone et al. (2018)	2	1	2	2	0	1	0	0	8

### 3.3 Primary outcomes

**FIGURE 2:**
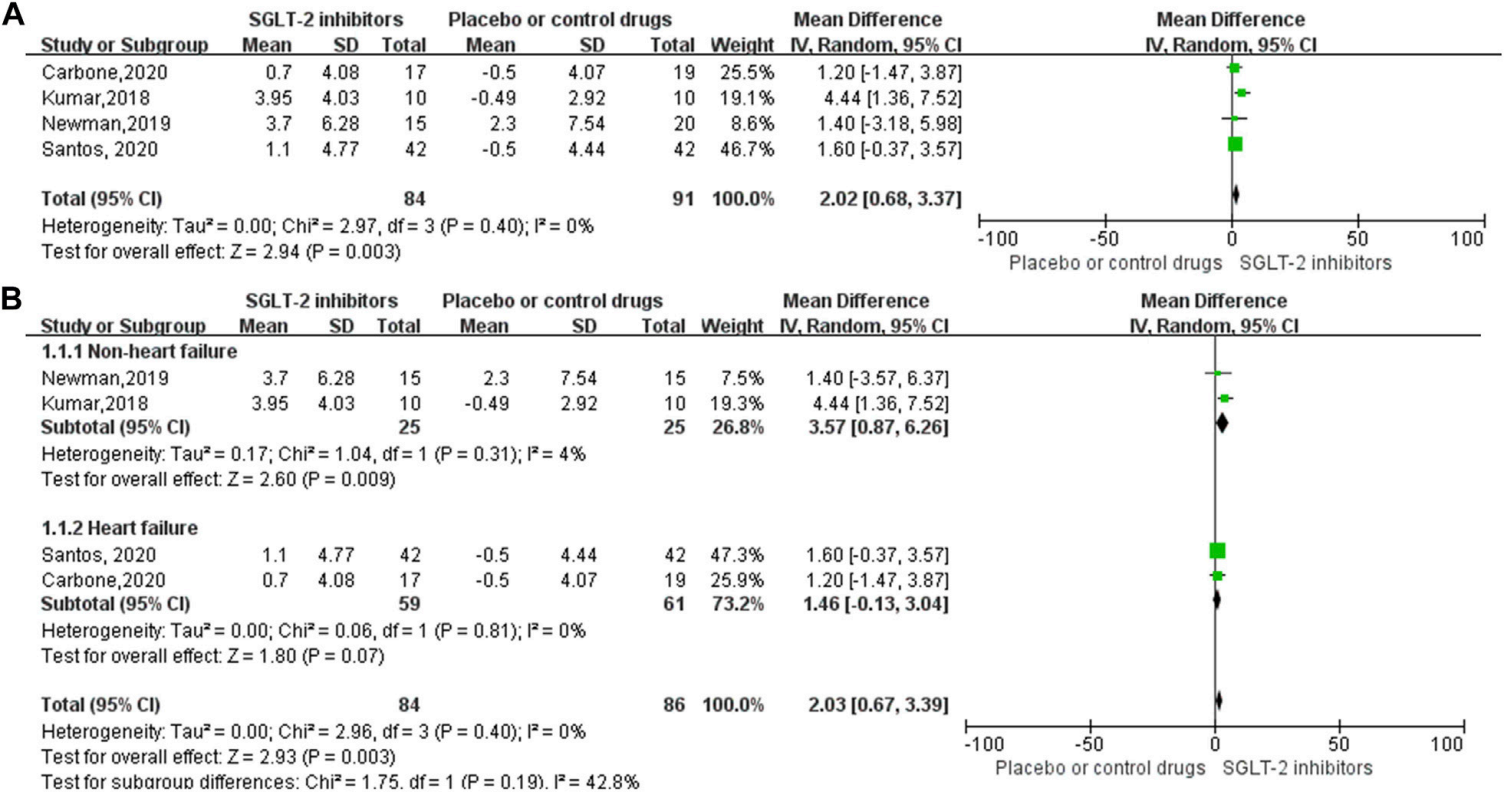
Forest plot for the VO_2_peak (RCTs). **(A)** Forest plot for the VO_2_peak from four RCT studies; SGLT-2 inhibitor group compared with the placebo or control drug group. **(B)** Forest plot of the subgroup analyses of VO_2_peak, according to the inclusion of the population, whether combined with heart failure. The subgroup was divided into two groups: non-heart failure or heart failure. SD, standard deviation; 95% CI, 95% confidence intervals; IV, inverse variance.

**FIGURE 3:**
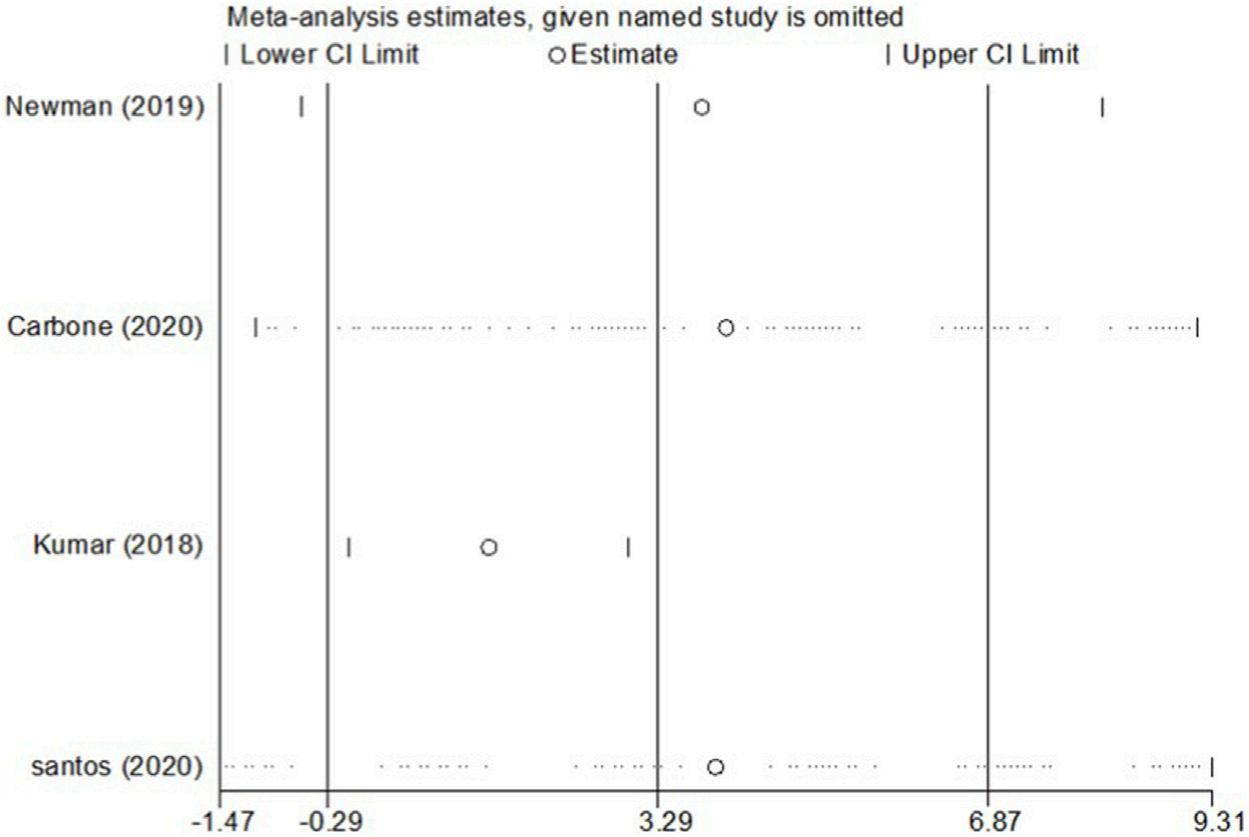
Sensitivity analysis for the effect size of VO_2_peak. Sensitivity analysis was conducted by removing each research individually to evaluate the quality and consistency of the outcomes. Removing each research individually did not change the statistical confidence interval.

**FIGURE 4:**
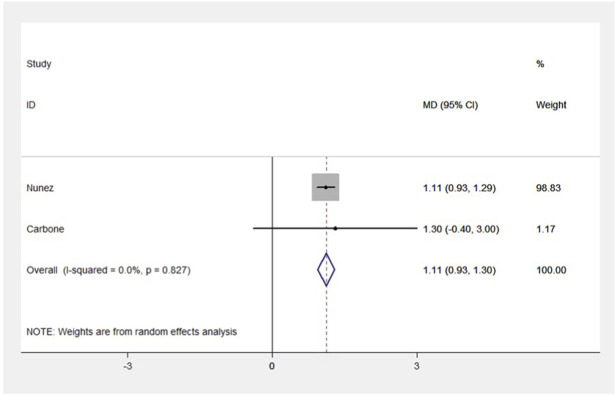
Forest plot for VO_2_peak (non-RCTs). The forest plot for VO_2_peak from two single-arm studies: pre- and post-intervention control. MD, weight mean difference; 95% CI, 95% confidence intervals.

Four RCT studies included the results of VO_2_peak, and the merge results showed that SGLT-2 inhibitors could significantly increase the VO_2_peak level (WMD, 2.02 ml kg^−1^ min^−1^, 95% CI: 0.68–3.37, *p* = 0.03, Figure 2A) in the obese population and patients with stable chronic HF, and high risk for CVD individuals with or without T2DM, which had beneficial effects on promoting cardiopulmonary fitness. The heterogeneity was very low (*p* = 0.40; I^2^ = 0%) as sensitive analysis was used to investigate the potential sources of heterogeneity and deleted any of the included studies for sensitive analysis, which did not substantially change the outcomes (Figure 3). Subgroup analysis showed that SGLT-2 inhibitors improve the VO_2_peak in patients without HF (WMD, 3.57 ml kg^−1^ min^−1^, 95% CI: 0.87–6.26, *p* = 0.009; I^2^ = 4%, *p* = 0.31) more than HF patients (WMD, 1.46 ml kg^−1^ min^−1^, 95% CI: −0.13–3.04, *p* = 0.07; I^2^ = 0%, *p* = 0.81, Figure 2B). In addition, the merge of single-arm studies also indicated that empagliflozin could improve VO_2_peak (MD, 1.11 ml kg^−1^ min^−1^, 95% CI: 0.93–1.30, *p* = 0.000) of T2DM patients with chronic HF, and the heterogeneity was very low (I^2^ = 0%, *p* = 0.827), as shown in the specific data information in Figure 4. Therefore, the aggregate results of those RCT and non-RCT studies indicated that SGLT-2 inhibitors could improve the cardiopulmonary fitness parameter of VO_2_peak in the obese population, patients with stable chronic HF, and high risk for CVD individuals with or without T2DM.

### 3.4 Secondary outcomes

**FIGURE 5:**
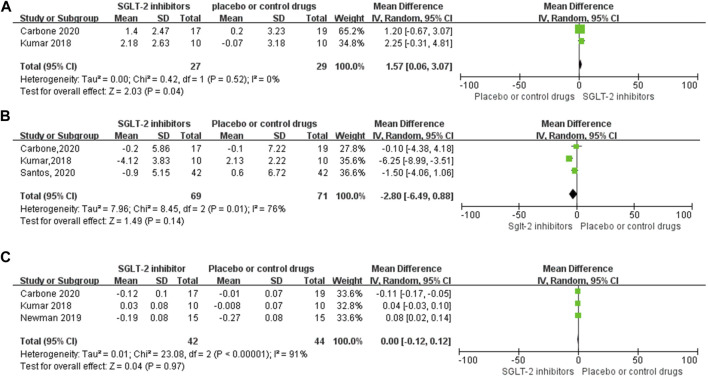
Forest plot for VAT, VE-VCO2 slope, and RER. **(A–C)** Forest plot for VAT, VE-VCO2 slope, and RER from four RCT studies, respectively; SGLT-2 inhibitor group compared with the placebo or control drug group. SD, standard deviation; 95% CI, 95% confidence interval; IV, inverse variance.

Four RCT studies also investigated the effect of SGLT-2 inhibitors on the RER, VE/VCO_2_ slope (minute ventilation/carbon dioxide production), VAT (ventilator anaerobic threshold), and other exercise capacity parameters. The meta-analysis results indicated that SGLT-2 inhibitors significantly improved the VAT (WMD, 1.57 ml kg^−1^ min^−1^, 95% CI: 0.06–3.07, *p* = 0.04, Figure 5A) level compared to the control group, and the heterogeneity was very low (*p* = 0.52; I^2^ = 0%). However, no significant changes were observed between the VE/VCO_2_ slope value (WMD, −2.80, 95% CI: −6.49–0.88, *p* = 0.14, Figure 5B) and RER (WMD, 0, 95% CI: −0.12–0.12, *p* = 0.97, Figure 5C), respectively. Both the VE/VCO_2_ slope (*p* = 0.01; I^2^ = 76%) and RER (*p* < 0.00001; I^2^ = 91%) had very high heterogeneity, as shown in the specific data information in Figures 5A–C.

## 4 Discussion

This meta-analysis preliminarily indicated that SGLT-2 inhibitors could increase the VO_2_peak and the VAT level of the obese population, patients with stable chronic HF, and high risk for CVD individuals with or without T2DM. However, SGLT-2 inhibitors did not significantly affect the exercise parameters of the VE/VCO_2_ slope and RER. SGLT-2 inhibitors improved the VO_2_peak and VAT of stable chronic HF patients with or without T2DM, which has beneficial effects on promoting cardiopulmonary fitness to help them improve prognosis. It was the first meta-analysis that provided new evidence to demonstrate that SGLT-2 inhibitors could increase the exercise capacity of stable chronic HF and high risk for CVD individuals with or without T2DM, which was inconsistent with metformin affecting the VO_2_peak of T2DM patients (Das et al., 2018a). Some previous studies illustrated that metformin attenuated peak aerobic capacity or did not significantly affect the VO_2_peak (Braun et al., 2008; Boulé et al., 2011). Moreover, increasing evidence demonstrated that metformin attenuated favorable physiological adaptations to exercise, which had adverse interactions between metformin and exercise. For instance, metformin attenuated the beneficial effect of exercise-inducing improvement in insulin sensitivity (Sharoff et al., 2010) and attenuated some beneficial effects of exercise on the risk factors of CVD (Malin et al., 2013). Therefore, SGLT-2 inhibitors improved cardiopulmonary fitness, which played an important supplementary role in the treatment of T2DM patients with metformin.

A previous study showed that aerobic exercise capacity negatively correlated with all-cause mortality and reduction of exercise tolerance, which is an independent predictor of the poor prognosis of individuals with HF (Goda et al., 2011). Our meta-analysis showed a 2.02 ml kg^−1^·min^−1^ of improvement in peak VO_2_ in the SGLT-2 inhibitor intervention group and preliminarily demonstrated that SGLT-2 inhibitors could increase the peak VO_2_ of T2DM or patients with stable chronic HF and improve exercise tolerance, which will play a critical role in improving the quality of life and reducing the risk of death in stable chronic HF patients with or without T2DM. Unfortunately, fewer RCT trials with limited sample capacity were included in our analysis. Meanwhile, in trials with an inadequate level of blinding in the original literature, some studies are non-placebo-controlled (Kumar et al., 2018; Carbone et al., 2020a), which could lead to a high risk of bias to show exaggerated treatment effects. Hence, it is necessary to design larger, high-quality RCTs and prospective cohorts to demonstrate whether SGLT-2 inhibitors can improve the peak VO_2_ of T2DM combined with chronic HF patients in the future. Moreover, the studies included in our meta-analysis did not widely report the outcome of lean peak VO_2_ and the percentage of predicted maximal exercise oxygen consumption, which were more sensitive than peak VO_2_ for predicting prognosis in patients with HF (Aaronson and Mancini, 1995; Osman et al., 2000). Particularly in subjects with obesity, CRF was underestimated if the peak VO_2_ was adjusted by total body mass (Del Buono et al., 2019; Carbone et al., 2020b). Because most T2DM patients are always associated with obesity, the peak VO_2_ adjusted by lean body mass might be a stronger prognosticator than the VO_2_peak. On the contrary, the percentage of predicted maximal exercise oxygen consumption rather than an absolute value may be a better predictor of survival for female patients with HF (Aaronson and Mancini, 1995). Therefore, for the outcome of lean peak VO_2_, the percentage of predicted maximal exercise oxygen consumption could be applied to research the effect of SGLT-2 inhibitors on the exercise capacity or CRF of T2DM patients combined with HF in the future.

Cardio-pulmonary exercise testing (CPET), a method for assessing exercise capacity, was regarded as the “gold standard” that evaluated peak VO_2_ as effort-dependent. The treadmill protocol could overestimate VO_2_ peak capacity up to approximately 10% against the cycle ergometer protocol (Busque et al., 2022). The studies included in our meta-analysis used the treadmill and cycle ergometer to evaluate the exercise capacity parameters, and the heterogeneity of the evaluation protocol could be difficult to reflect actual exercise capacity. In contrast, VAT was not effort-dependent. VAT was also demonstrated to be a stronger prognosticator for HF patients than VO_2_peak by Del Buono et al. (2019). This meta-analysis preliminarily indicated that SGLT-2 inhibitors improved VAT in T2DM patients combined with HF, which was consistent with the VAT of healthy volunteers affected by metformin (Das et al., 2018a). Because of the severe limits of exercise tolerance in patients with chronic HF, they rarely participated in high-intensity activities during their daily life. VAT could quantify the ability to sustain submaximal physical activities, approximated to the levels associated with daily life activities in patients with HF. Therefore, SGLT-2 inhibitors improved VAT and prognosis in T2DM patients combined with HF. Our meta-analysis showed that both parameters of VO_2_peak and VAT are consistent with increasing aerobic tolerance capacity. The mechanisms by which SGLT-2 inhibitors improved exercise tolerance capacity of T2DM patients combined with HF were multifactorial and complex and had not yet been completely elucidated. The current mechanisms were probably associated with the effect of SGLT-2 inhibitors to elevate hematocrit and erythropoietin to increase oxygen delivery, improve mitochondrial fatty acid oxidation in skeletal muscle, lose weight and increase synthesis of ketone bodies, and convert energy metabolism substrate from glucose to fatty acid oxidation for utilization by the heart (Cowie and Fisher, 2020).

RER was defined as the ratio of CO_2_ production to O_2_ intake during metabolism, which was used to calculate the relative energy consumption of carbohydrates and lipids (Pendergast et al., 2000). A high RER level showed that carbohydrates were mainly used, whereas a low RER indicated lipid oxidation. Our study showed that SGLT-2 inhibitors did not significantly affect the RER among the individuals compared with the placebo or control drug group, which was consistent with metformin affecting RER of T2DM patients (Das et al., 2018a). However, this outcome had very high heterogeneity. A study showed that there was a lower RER at the trial deadline in the canagliflozin group, which might be related to the imbalance at baseline (Carbone et al., 2020a). Notably, some evidence showed that SGLT-2 inhibitors reduced the tendency of the RER value before and after the intervention, but they had no negative effects on the VO_2_peak (Carbone et al., 2018; Newman et al., 2019; Carbone et al., 2020a). Whether SGLT-2 inhibitors could reduce RER by increasing lipid oxidation remains unknown and needs further research. Furthermore, the effort-independent CPX variable and VE/VCO_2_ slope did not change significantly by SGLT-2 inhibitors in our meta-analysis. This result was related to few included studies with a higher heterogeneity. Although the meta-analysis results showed no significant statistical difference in the changes in the VE/VCO_2_ slope, studies of Carbone, Kumar, and Santos showed that SGLT-2 inhibitors reduce the VE/VCO_2_ slope, especially by 15.8% in the inhibitor group of Kumar’s research (Kumar et al., 2018; Carbone et al., 2020a; Santos-Gallego et al., 2020), which was consistent with previous reports stating that beta-blockers and ACE inhibitors could significantly improve VE/VCO_2_ slope in patients with HF (Chaudhry et al., 2009). Therefore, according to the aforementioned research evidence, SGLT-2 inhibitors helped patients with T2DM combined with HF improve CRF, which had an important clinical application value and basis. It is necessary to further explore the effect of SGLT-2 inhibitors on CRF in patients with T2DM combined with HF and provide more clinical evidence with SGLT-2 inhibitors to prevent T2DM patients combined with HF.

Regular exercise was recommended as an important treatment and prevention for T2DM patients, and the exercise combined with the antidiabetic agent was prescribed in clinics to control glucose homeostasis. Noteworthy, previous studies showed that metformin, in GLP-1 receptor antagonism, had potentially adverse interactions with exercise (Myette-Côté et al., 2016; Jørgensen et al., 2017). The influence of SGLT-2 inhibitors on the physiological adaptation to exercise is not determined at present. A previous study preliminarily showed that SGLT-2 inhibitors combined with endurance exercise did not weaken or enhance the exercise capacity of obese people. On the contrary, the improvements in insulin sensitivity induced by long-term exercise training were inhibited by SGLT-2 inhibitors (Newman et al., 2019). However, the results of animal experiments indicated that SGLT-2 inhibitors improved glucose tolerance and exercise endurance capacity in a rodent model of T2DM (Linden et al., 2019). At present, there is insufficient evidence that SGLT-2 inhibitors interacted adversely with exercise-induced beneficial physiological adaptation. Whether SGLT-2 inhibitors have negative influences on exercise-induced beneficial physiological adaptation in patients with T2DM needs further research. Therefore, as with other antidiabetic agents, whether SGLT-2 inhibitors combined with exercise could lead to some adverse physiological adaptation should be considered by clinicians. Meanwhile, the mechanism of these co-prescribed therapies for improving the aerobic and anaerobic capacity in patients with T2DM combined with HF needs further discussion. The experimental research on SGLT-2 inhibitors on the response to resistance training intervention had not been conducted yet, and there was no doubt that it would be new highlight research that SGLT-2 inhibitors combined with exercise prescription therapies could improve chronic disease management.

As far as we know, this is the first meta-analysis to indicate that SGLT-2 inhibitors could improve some parameters of exercise capacity in stable chronic HF patients with or without T2DM or the obese population. However, there were certain limitations in this study. Primarily, fewer RCT trials with limited sample capacity were included in our analysis, and a medium-to-high risk of bias was observed in all the included studies, which resulted in low-grade evidence for the outcomes. Secondarily, outcomes of RER and VE/VCO_2_ slope have higher heterogeneity. Furthermore, a wide variety of SGLT-2 inhibitors approved for use currently, such as empagliflozin, canagliflozin, and dapagliflozin, lack adequate studies to compare different kinds of SGLT-2 inhibitors’ effect on the exercise capacity of T2DM patients. The doses of the different kinds of SGLT-2 inhibitors used were not uniform; there were multiple differences. The duration of SGLT-2 inhibitor therapy in different studies also showed a significant difference (1–6 months). Due to unavailable data, the duration-based subgroup analysis and the dose of SGLT-2 inhibitor therapy were not probably performed. Therefore, it was necessary to demonstrate the effect of the dose and duration of SGLT-2 inhibitors therapy on the exercise capacity of T2DM patients in the future. In addition, it was the only therapy in which exercise training could effectively improve CRF in patients with heart failure with preserved ejection (HFpEF) (Haykowsky et al., 2012), whereas previous studies indicated that most of the therapy drugs for HF were ineffective in improving exercise capacity in patients with HFpEF currently (Del Buono et al., 2019). However, no studies have reported whether SGLT-2 inhibitor therapy could effectively enhance the exercise capacity of patients with HFpEF. Furthermore, the patients in our study who took many other types of CVD therapy drugs and glucose-lowering agents, except for SGLT-2 inhibitors, such as β-adrenergic receptor blockers, statins, loop diuretics, and DPP4 inhibitors, might interfere with exercise capacity (Masutani et al., 2013; Bahls et al., 2017; Mandai et al., 2017). As drugs commonly used for HF treatment, beta-blockers and ACE inhibitors have been proven to improve peak VO_2_ and reduce VE/VCO_2_ for patients with HF (Chaudhry et al., 2009). Multiple-drug therapy could be an important selecting bias, and the mixed physiological effects of multiple drugs could result in heterogeneous outcomes and a lower grade of evidence.

## 5 Conclusion

Notwithstanding these limitations, this meta-analysis has preliminarily demonstrated that SGLT-2 inhibitors can increase the peak VO_2_ and VAT of the obese population, patients with stable chronic HF, and high risk for CVD individuals with or without T2DM and improve the exercise tolerance capacity, which has beneficial effects on promoting cardiopulmonary fitness to help T2DM combined with HF patients improve prognosis and reduce the all-cause mortality risk. The unique cardiovascular protective benefits of SGLT-2 inhibitors made it a broad application prospect. However, due to the limited samples and few RCTs, it is necessary to interpret and apply the outcomes of this research cautiously. In addition, SGLT-2 inhibitors could reduce weight, thus increasing the relative values of maximal oxygen uptake. The influence of variances, such as body weight and lean weight, should be considered, and the outcomes of lean peak VO_2_ could be applied to research the effects of SGLT-2 inhibitors on the exercise capacity or CRF of T2DM patients combined with HF in the future. Meanwhile, in order to demonstrate the effects of SGLT-2 inhibitors on exercise capacity and provide patients with safer choices, the efficacy and safety of the combined application of SGLT-2 inhibitors and exercise therapy in clinical applications need further confirmation *via* evidence-based studies.

## Data Availability

The original contributions presented in the study are included in the article/Supplementary Material. Further inquiries can be directed to the corresponding author.
