# Supplementary material for: The effect of SGLT-2 inhibitors on cardiorespiratory fitness capacity: A systematic review and meta-analysis
Source: Front Physiol. 2023 Jan 10;13:1081920. doi: 10.3389/fphys.2022.1081920 (PMC9871354; doi:10.3389/fphys.2022.1081920)
Supplement: Supplementary file 2 [file Table1.docx]

**Supplementary material**

**Search strategy:**

Pubmed

#1 "Single-Blind Method"[Mesh] OR "Double-Blind Method"[Mesh] OR "Randomized Controlled

Trials as Topic"[Mesh] OR "Randomized Controlled

Trial" [Publication Type] OR "Intention to Treat Analysis"[Mesh] OR "Controlled Clinical Trials as

Topic"[Mesh] OR "Clinical Trials as Topic"[Mesh]

OR "Clinical Trial" [Publication Type] OR randomized controlled trial[Publication Type]

#2 "random*"[Text Word] OR allocation[Text Word] OR "random allocation"[Text Word] OR

placebo[Text Word] OR single blind[Text Word] OR double

blind[Text Word] OR "randomized controlled trial*"[Text Word] OR RCT[Text Word]

#3 #1 OR #2

#4 animals NOT humans

#5 #3 NOT #4

#6 SGLT2[Title/abstract] OR “SGLT2 inhibitor”[Title/abstract] OR “Sodiumglucose cotransporter 2 inhibitor”[Title/abstract] OR dapagliflozin [Title/abstract] OR canagliflozin [Title/abstract] OR empagliflozin [Title/abstract] OR ipragliflozin [Title/abstract] OR luseogliflozin [Title/abstract] OR tofogliflozin [Title/abstract]

#7“cardiorespiratory fitness”[Title/abstract] OR “cardiopulmonary endurance”[Title/abstract] OR endurance[Title/abstract] OR exercise[Title/abstract] OR “exercise capacity”[Title/abstract] OR “exercise tolerance”[Title/abstract] OR aerobic[Title/abstract] OR “aerobic exercise”[Title/abstract] OR oxygen[Title/abstract] OR “oxygen consumption”[Title/abstract] OR “VO_2_”[Title/abstract] OR “peak VO_2_”[Title/abstract] OR “VO_2max_”[Title/abstract] OR “VO_2peak_”[Title/abstract]

#8 #5 AND #6 AND #7

Web of sci

#1 TS=("random*" OR allocation OR "random allocation" OR placebo OR single blind OR single

blind method OR double blind OR double blind method OR "randomized controlled trial*" OR

"randomised controlled trial*" OR "RCT" OR "clinical trial*")

#2 TS=(SGLT2 OR “SGLT2 inhibitor” OR “Sodiumglucose cotransporter 2 inhibitor” OR dapagliflozin OR canagliflozin OR empagliflozin OR ipragliflozin OR luseogliflozin OR tofogliflozin)

#3 Ts=(“cardiorespiratory fitness” OR “cardiopulmonary endurance” OR endurance OR exercise OR “exercise capacity” OR “exercise tolerance” OR aerobic OR “aerobic exercise” OR oxygen OR “oxygen consumption” OR “VO2” OR “peak VO2” OR “VO2max” OR “VO2peak”)

#4 #3 AND #2 AND #1

Databases= WOS, KJD, RSCI, SCIELO Timespan=All years Search language=Auto

Search Strategy for Cochrane Library

#1 "random*" or allocation or "random allocation" or placebo or single blind or double blind or "randomized controlled trial*" or RCT or "clinical trial*" in Title Abstract Keyword

#2 SGLT2 OR “SGLT2 inhibitor” OR “Sodiumglucose cotransporter 2 inhibitor” OR dapagliflozin OR canagliflozin OR empagliflozin OR ipragliflozin OR luseogliflozin OR tofogliflozin in Title Abstract Keyword

#3 “cardiorespiratory fitness” OR “cardiopulmonary endurance” OR endurance OR exercise OR “exercise capacity” OR “exercise tolerance” OR aerobic OR “aerobic exercise” OR oxygen OR “oxygen consumption” OR “VO2” OR “peak VO2” OR “VO2max” OR “VO2peak” in Title Abstract Keyword

#4 #1 and #2 and #3

Search Strategy for EMBASE

#1 'randomization'/exp OR 'placebo'/exp OR 'placebo effect'/exp OR 'single

blind procedure'/exp OR 'double blind procedure'/exp OR 'randomized

controlled trial'/exp OR 'randomized controlled trial (topic)'/exp OR 'controlled

clinical trial'/exp OR 'controlled clinical trial (topic)'/exp OR 'clinical trial'/exp

OR 'clinical trial (topic)'/exp

#2 (((((random*:ab,ti OR allocation:ab,ti OR 'random allocation':ab,ti OR placebo:ab,ti OR single) AND blind:ab,ti OR double) AND blind:ab,ti OR randomized) AND controlled AND trial*:ab,ti OR randomized) AND controlled AND trial*:ab,ti OR rct:ab,ti OR clinical) AND trial*:ab,ti

#3 #1 OR #2

#4 sglt2 AND ab,ti OR “sglt2 inhibitor”:ab,ti OR “sodiumglucose cotransporter 2 inhibitor”:ab,ti OR dapagliflozin:ab,ti OR canagliflozin:ab,ti OR empagliflozin:ab,ti OR ipragliflozin:ab,ti OR luseogliflozin:ab,ti OR tofogliflozin:ab,ti

#5 “cardiorespiratory fitness”:ab,ti OR “cardiopulmonary endurance”:ab,ti OR endurance:ab,ti OR exercise:ab,ti OR “exercise capacity":ab,ti OR “exercise tolerance”:ab,ti OR aerobic:ab,ti OR “aerobic exercise”:ab,ti OR oxygen:ab,ti OR “oxygen consumption”:ab,ti OR “VO2”:ab,ti OR “peak VO2”:ab,ti OR “VO2max”:ab,ti OR “VO2peak”:ab,ti

#6 #3 and #4 and #5

Search Strategy for EBSCO CINAHL

S1 MH "random assignment" OR placebos OR "placebo effect" OR "single-blind studies" OR "double-blind studies" OR "randomized controlled trials" OR "clinical trials"

TX ( random$ OR allocation OR "random allocation" OR placebo$ OR single blind OR double blind OR "randomi? ed controlled trial*" OR "controlled clinical trial*" OR "RCT" OR "clinical trial*" )

S2 PT( randomized controlled trial OR "clinical trial*")

S3 S1 OR S2

S4 AB SGLT2 OR “SGLT2 inhibitor” OR “Sodiumglucose cotransporter 2 inhibitor” OR dapagliflozin OR canagliflozin OR empagliflozin OR ipragliflozin OR luseogliflozin OR tofogliflozin

S5 AB “cardiorespiratory fitness” OR “cardiopulmonary endurance” OR endurance OR exercise OR “exercise capacity” OR “exercise tolerance” OR aerobic OR “aerobic exercise” OR oxygen OR “oxygen consumption” OR “VO2” OR “peak VO2” OR “VO2max” OR “VO2peak”

S6 S3 AND S4 AND S5
